# Evaluation of dithiothreitol-oxidizing capacity (DOC) as a serum biomarker for chronic hepatitis B in patients exhibiting normal alanine aminotransferase levels: a pilot study towards better monitoring of disease

**DOI:** 10.1016/j.eclinm.2021.101180

**Published:** 2021-10-30

**Authors:** Lumin Yang, Yafei Zhang, Ke Zhang, Zhongping Liu, Tengfei He, Xiaowei Zheng, Lei Li, Elias S J Arnér, Zhenhua Zhang, Jinsong Zhang

**Affiliations:** 1 State Key Laboratory of Tea Plant Biology and Utilization, School of Tea & Food Science, Anhui Agricultural University, Hefei, Anhui, China; 2 Department of Infectious Diseases and Institute of Clinical Virology, The Second Hospital of Anhui Medical University, Hefei, Anhui, China; 3 Department of Infectious Disease, Anhui Provincial Hospital of Anhui Medical University, Hefei, Anhui, China; 4 Division of Biochemistry, Department of Medical Biochemistry and Biophysics, Karolinska Institutet, Stockholm, Sweden and Department of Selenoprotein Research, National Institute of Oncology, Budapest, Hungary.

**Keywords:** dithiothreitol-oxidizing capacity, alanine aminotransferase, sulfhydryl oxidase, chronic liver diseases, complementary biomarker

## Abstract

**Background:**

Alanine aminotransferase (ALT) is the most commonly used serum biomarker for chronic liver diseases (CLDs) but may not accurately reflect hepatic disorders and easily underestimates hepatic fibrosis. The previously revised upper limit of normal (ULN) of ALT (19 U/L for women and 30 U/L for men) increases its sensitivity but yields higher numbers of false-positives. Moreover, CLDs patients with ALT lower than the revised ULN may nonetheless have progression of disease. Therefore there is a need of novel biomarkers to complement the use of ALT. Here we have evaluated measurements of serum dithiothreitol-oxidizing capacity (DOC) in cohorts of chronic hepatitis B patients with different stages of disease as an exploratory pilot study for this purpose.

**Methods:**

Serum samples obtained from healthy persons and from chronic hepatitis B patients with normal ALT values were used for sensitivity evaluation. The hepatitis B patients encompassed end-stage liver diseases (ELD), chronic hepatitis B (CHB), CHB with persistently normal ALT (CHB-P) and inactive carriers (ICs). Sensitivity was also evaluated with samples from patients with other diseases. The study period was March 2018 to December 2020.

**Findings:**

DOC was found to be a robust biomarker that may become complementary to ALT measurements, especially in patients displaying low ALT levels. ROC analyses indicated that the AUC values of DOC reached 0.983 and 0.956 in ELD and CHB patients exhibiting normal ALT levels, respectively. Importantly, the AUC values of DOC reached 0.852 and 0.844 in CHB-P patients and ICs, respectively. Such AUC values permit screening and continued monitoring, corresponding to over 30% and 50% sensitivity with 99% and 95% specificity for CHB-P and ICs, respectively. DOC was also significantly correlated with indicators for fibrosis, assessing both APRI (Pearson r = 0.4905, P < 0.0001) and FIB-4 (Pearson r = 0.4421, P < 0.0001). Surprisingly, the AUC values of DOC in the hepatitis B patients with ALT levels lower than the revised ULN were not compromised. In examined non-liver diseases, DOC was low and normal, including in patients with acute myocardial infection displaying increased ALT levels.

**Interpretations:**

The results suggest that DOC can be promising as a complementary biomarker used in addition to ALT for monitoring of disease in chronic hepatitis B patients, especially when ALT levels are normal. DOC should be further evaluated for possible clinical use as biomarker also in other CLDs.

**Funding:**

This study was funded by the National Natural Science Foundation of China (Grant numbers: 31771971 and 32001013).

**Research in context**

**Evidence before this study**

Alanine aminotransferase (ALT) is the most commonly used serum biomarker for clinical monitoring of liver disease despite evidence of low sensitivity and low specificity. Rather recently proposed revisions in the reference interval for normal ALT levels may further impair its use as a biomarker, as a decreased upper normal limit yields higher numbers of false positives, while still showing too low sensitivity for use in cases of liver fibrosis. Thus, there is a major and widely acknowledged need for new or additional biomarkers to aid facile assessment and monitoring of liver disease.

**Added value of this study**

Here we present results from an exploratory pilot study suggesting that the assessment of sulfhydryl oxidase activities in serum, determined through a new activity assay hereby named dithiothreitol-oxidizing capacity (DOC), can provide a facile and reliable biomarker that may be significantly more robust than ALT for assessment of disease in different cohorts of chronic hepatitis B patients, especially in those displaying low or normal ALT levels. It also correlated well with markers for liver fibrosis. We suggest that DOC can be evaluated as an additional biomarker together with ALT that should be of special diagnostic value in monitoring of patients displaying normal ALT levels.

**Implications of all the available evidence**

Based upon our findings we propose that serum DOC should be further evaluated for potential use in clinical monitoring of disease progression in chronic hepatitis B, and possibly also in other liver diseases.

Alt-text: Unlabelled box

## 1 Introduction

Chronic liver disease (CLD) is highly prevalent around the world and considered a major public health problem. Current estimates suggest that 844 million people have CLDs, a higher number than for diabetes (422 million), cardiovascular (540 million) or pulmonary diseases (650 million) [1]. The most commonly used serum biomarker for CLD is alanine aminotransferase (ALT). Unfortunately, ALT serum levels do not well correlate with severity of disease and many patients with normal ALT levels are at risk of having ongoing non-discovered hepatic inflammation and fibrosis [2], [3], [4], [5], [6]. Therefore the upper limit of normal (ULN) for ALT, adjusted for sex differences (19 U/L for women and 30 U/L for men), was proposed to be lowered in order to increase sensitivity [2,7]. However, a revised lower ULN has not been widely adopted, as it may yield rather high numbers of false-positives [8,9]. Furthermore, CLD patients with ALT even below the revised ULN may still be severely affected by disease [10] and fibrosis was found in 27.8% of chronic hepatitis B (CHB) patients with ALT lower than the revised ULN [10]. Patients with ALT less than half the value of ULN may still have non-alcoholic steatohepatitis with significant fibrosis [5]. Therefore, new sensitive yet robust serum biomarkers for facile assessment of disease are urgently needed to complement the use of ALT, especially in patients presenting low ALT levels. Here we evaluated a new biomarker for this purpose with serum from cohorts of chronic hepatitis B patients.

Currently, approximately 3.5% of the global population is estimated to be chronically infected with hepatitis B virus (HBV) [11]. HBV infection causes various outcomes, ranging from inactive carriers (ICs) to end-stage liver disease (ELD) that includes cirrhosis, liver failure and hepatocellular carcinoma. ICs have normal ALT levels [12], [13], [14]. Many chronic hepatitis B (CHB) patients also exhibit persistently normal ALT levels (referred to as CHB-P herein) [4,10,15]. ELD patients may also show intermittently normal ALT levels, despite severe liver pathology [16,17]. The urgency of better biomarkers for assessment of disease in patients infected with HBV as well as their variable clinical presentations make this patient cohort highly suitable for evaluation of new biomarkers for assessment of liver disease.

We have previously found that serum TXNRD activity increased upon liver injury in mice [18]. Subsequently we indentified that the enzymatic TXNRD activity is counteracted by quiescin Q6 sulfhydryl oxidase 1 (QSOX1) also present in either human or mouse serum [19]. QSOX1 accounts for the major part of sulfhydryl oxidases (SOX) in serum, since inhibitory monoclonal antibodies specific for human QSOX1 largely inhibited the activity opposing the TXNRD measurements [19]. A sensitive plate-reader assay for determination of serum QSOX1 activity, based upon fluorescence measurements of Amplex UltraRed-hydrogen peroxide complexes, has been developed [20]. That method for SOX activity determinations is hence based upon detection of the enzymatic reaction product (H_2_O_2_, hydrogen peroxide). Alternatively, we found that measurements of the disappearance of an artificial thiol substrate, dithiothreitol (DTT), here referred to as DTT-oxidizing capacity (DOC), could also be used to assess the total thiol oxidation activity in serum [19]. The DOC assay is colorimetric, easy to perform, stable and inexpensive, making its methodological features attractive for clinical use. Thus, we here evaluated the potential of using either SOX or DOC activity determinations in serum as biomarkers for disease in CLDs. We focused on the use of these assays in cohorts of chronic hepatitis B patients having normal ALT levels to assess whether they are able to complement the drawbacks of low ALT levels as shown above. The results suggest that DOC, but not SOX, activity measurements can provide informative value as an additional biomarker for CLD that can become complementary to ALT determinations, especially in patients displaying low ALT values in spite of ongoing disease.

## 2 Materials and Methods

### 2.1 Chemicals

DTT, 5,5-dithiobis-2-nitrobenzoicacid (DTNB), HEPES, Tween-80, guanidine hydrochloride and horseradish peroxidase (HRP) were all obtained from Sigma (St. Louis, MO, USA). Amplex UltraRed was purchased from Life Technologies (Carlsbad, CA, USA). Other chemicals were of the highest grade available.

### 2.2 Patients and specimens

For this study, we wished to obtain samples from local hospitals of as many CLD patients as practically possible. In total, 2251 serum samples from adult donors were used in this cross-sectional study. To evaluate sensitivity of the examined biomarkers in CLDs, we recruited 1693 CLD patients from four cohorts of different clinical presentations of HBV-infected patients: 757 ELD patients under drug treatment (419 cases with intermittently normal ALT and 338 with abnormal ALT); 511 CHB patients under drug treatment (196 with intermittently normal ALT and 315 with abnormal ALT); 217 CHB-P patients; 208 ICs. The definitions of studied cohorts followed the guidelines regarding the management of chronic HBV infection [12], [13], [14]. Briefly, i) the ELD cohort was composed of HBV-associated decompensated cirrhosis, liver failure and hepatocellular carcinoma patients; ii) the CHB cohort was composed of HBV-associated chronic hepatitis patients; iii) the CHB-P cohort was composed of CHB patients under drug treatment and with persistently normal ALT for the last 12 months; and iv) the IC cohort was composed of inactive HBV carriers with normal ALT and without drug intervention. HBsAg was positive for more than 6 months in all groups. For the CHB patients, HBV DNA levels were above 2000 IU/mL before antiviral therapy. HBV DNA levels of ICs were under 2000 IU/mL. HBeAg was positive or negative in CHB and CHB-P patients, and was negative in ICs. To evaluate performance of the biomarkers in diseases not related to liver pathology, we recruited 77 patients with acute myocardial infarction (AMI), and 163 patients with other diseases than CLD or AMI, including 55 with stroke, 50 with diabetes, and 17 with pulmonary tuberculosis. As 318 healthy controls (HC), serum was obtained from overtly healthy blood donors having normal ALT, aspartate aminotransferase (AST), total bilirubin (TB) and direct bilirubin (DB). The number of persons with ages and gender distribution for HC donors and patients are given in Tables S1-S3. The study was approved by the ethics committees of Anhui Medical University, Anhui, China, and all study participants provided written informed consent. Samples without missing reference data of ALT, AST, TB and DB were randomly collected between March 2018 and December 2020 from three hospitals affiliated to the University in Anhui, China (the First Affiliated Hospital, the Second Hospital, and the Anhui Provincial Hospital). STARD (Standards for Reporting Diagnostic Accuracy) was used as reporting guideline.

### 2.3 Handling of blood samples

Venous blood samples were collected and centrifuged to obtain serum samples, which were stored at -80°C until analyses. The serum levels of ALT, AST, TB and DB were determined in the clinical routine laboratories of the local hospitals.

### 2.4 SOX activity assay and SOX units

The fluorescence-based assay for SOX activity was performed at 25°C according to the method of Israel et al. [20]. One unit (U) was defined as a fluorescence intensity increase by 1 per min and the resulting SOX activity was presented as U/μL serum.

### 2.5 DOC activity assay and DOC units

To measure the dithiol oxidation activities in serum (DOC), samples of serum (15 μL) diluted with saline (85 μL) were mixed with 50 μL of a reaction mixture containing 10 mM EDTA-Na_2_ and 1 mM DTT in HEPES (200 mM, pH 7.2). For background subtraction for each sample, serum (15 μL) and saline (85 μL) were also mixed with 50 μL of a mixture containing 10 mM EDTA-Na_2_ in HEPES without DTT. The difference of these paired serum tests represented total reaction mixture thiol levels in the presence of serum. As another control to measure thiol levels of the reaction mixture in the absence of serum, 100 μL saline was mixed with 50 μL HEPES (200 mM, pH 7.2, 10 mM EDTA-Na_2_), either with or without 1 mM DTT. The difference of these paired saline tests represented controls for total thiol levels in the absence of serum sample addition. These four reaction mixtures were made for each sample, and the DOC activity assay was thereupon performed by incubation at 37 °C for 15 min. Then 200 μL Tris-buffer (200 mM, pH 8.0) containing 6.6 M guanidine hydrochloride and 1 mM DTNB was added, in order to terminate the reaction by denaturation of all proteins by guanidine hydrochloride, and to determine total thiol contents by reaction with DTNB (releasing TNB^−^ anions with absorbance at 412 nm upon reaction with free thiols). After 5 min, and within 30 min following initiation of the assay, absorbance in each reaction was determined at 412 nm using a 96-well plate reader. The extent of thiol decrease in the serum sample during the assay, thus defining its DOC activity, was calculated using the following formula.

$$\frac{\text{Thiol}\;\text{levels}\;\text{in}\;\text{the}\;\text{absence}\;\text{of}\;\text{serum}\;-\text{Thiol}\;\text{levels}\;\text{in}\;\text{the}\;\text{presence}\;\text{of}\;\text{serum}}{\text{Thiol}\;\text{levels}\;\text{in}\;\text{the}\;\text{absence}\;\text{of}\;\text{serum}}\times \;100\%$$

If the thiol decrease in the assay exceeded 55%, serum was diluted for redetermination. One U of DOC was defined as 1% DTT (2% thiol) decrease during the assay duration of 15 min. DOC was then presented as U/μL serum.

### 2.6 Statistics

Receiver operating characteristics (ROC) curves and multivariate logistic regression analysis were analyzed by SPSS (version 17.0). Other analyses were performed by GraphPad Prism (version 5.0). Differences between two independent groups were tested with the Mann-Whitney U test if the data exhibited abnormal distribution as examined with the D'Agostino & Pearson omnibus normality test. Accordingly data are presented as median with 25% percentile and 75% percentile. Data are presented as mean ± range in case of two replicates. Coefficient of variation (CV) was calculated by standard deviation/mean. Goodness of fit in standard curve of SOX or DOC assay is presented as R^2^. Pearson correlation coefficient is presented as r. The more rigorous P value (less than 0.005) was considered statistically significant [21].

### 2.7 Role of funding source

The funders of the study had no role in study design, data collection, data analysis, data interpretation, or writing of the report. The corresponding authors had full access to all the data in the study and had final responsibility for the decision to submit for publication.

## 3 Results

### 3.1 Methodological validation of SOX and DOC assay performance

**Fig. 1:**
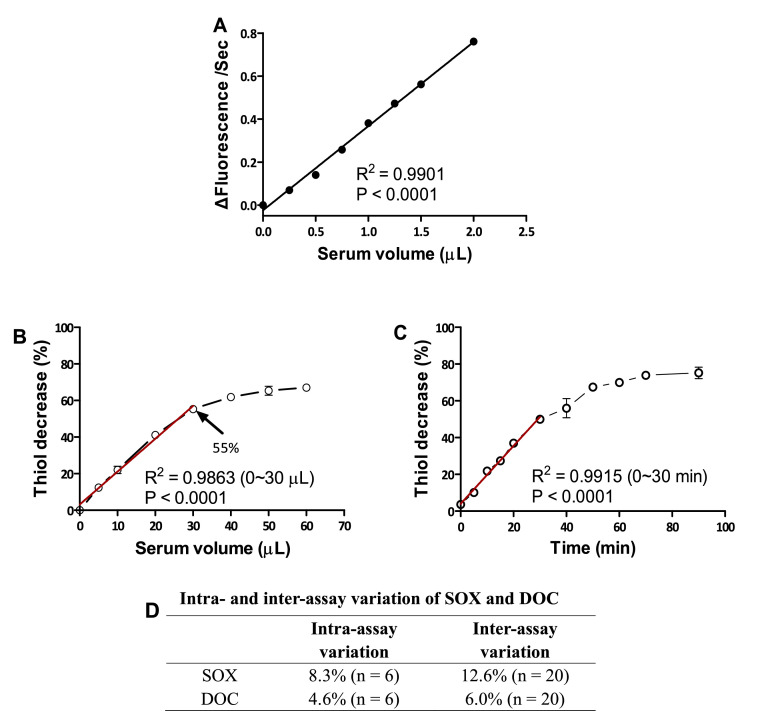
Characterization of SOX and DOC assay approaches. (A) Association of serum volume and ΔFluorescence/Sec following a 3-min reaction at 25 °C for determination of SOX activity. (B) Association of serum volume and thiol decrease during a 15-min reaction at 37 °C for determination of DOC. (C) Association of reaction time and thiol decrease during 15 min caused by 15 μL healthy pooled human serum at 37 °C in the assay of DOC. (D) Coefficient variation of intra- and inter-assay of SOX and DOC determinations. Data are presented as the mean of two replicates in A-C (error bar represents range). DOC, dithiothreitol-oxidizing capacity; SOX, sulfhydryl oxidases.

For SOX and DOC assay validations, a pooled healthy human serum sample was prepared. For SOX measurements, using up to 2 μL pooled serum, increases in fluorescence intensity rates during 3 minutes of assay linearly correlated with increased serum volumes (R^2^ = 0.9901, P < 0.0001) (Fig. 1A). For the readout of the DOC assay, thiol decrease during the assay linearly correlated (R^2^ = 0.9863, P < 0.0001) with increasing serum volumes up to 30 μL, where 55% of the thiols in the sample had decreased, whereafter saturation of the assay was reached (Fig. 1B). Thereby all consecutive DOC assays in this study were performed so that thiol decreases during the assay remained below 55%. Determining the time-dependent decrease of thiols in the assay using 15 μL pooled serum, a good linear correlation (R^2^ = 0.9915, P < 0.0001) was maintained up to 30-min of incubation (Fig. 1C). Based on this result, 15-min reactions were used for the remainder of this study. To assess the extent of intra-assay variation, the same pooled serum sample was used in six technical replicates, whereby the CV for the SOX assay and DOC assay were found to be 8.3% and 4.6%, respectively (Fig. 1D). For assessment of inter-assay variations, the same pooled serum was determined once daily at 20 different days; the CV of the SOX and DOC assay were in this case 12.6% and 6.0%, respectively (Fig. 1D). These results showed that the DOC assay accuracy and stability was higher than for the SOX determinations. For both assays, the pooled serum was however used as reference sample for all subsequent measurements of SOX and DOC activities in this study, with only data obtained from assays showing similar values for the reference sample (±5%) deemed reliable and used for the results as determined and analyzed herein.

### 3.2 DOC is a promising biomarker for disease monitoring in hepatitis B patients with normal ALT levels

**Fig. 2:**
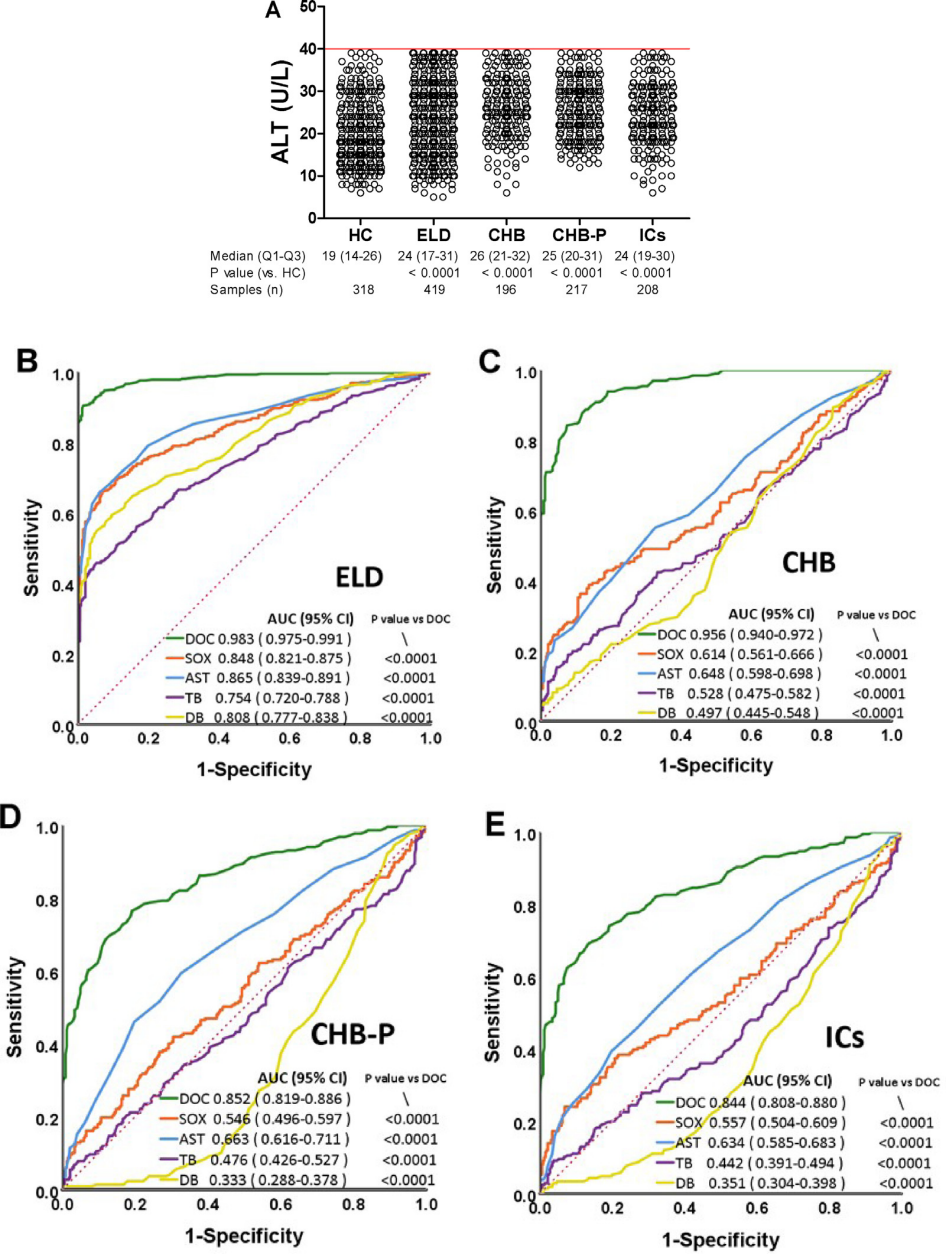
ROC analyses of serum biomarkers in chronic hepatitis B patients with normal ALT activity. (A) ALT levels in each tested group (Mann Whitney test). (B-E) ROC analyses of the ELD, CHB and CHB-P patients as well as ICs, respectively. AUC, area under the curve; ROC, receiver operating characteristic; DOC, dithiothreitol-oxidizing capacity; SOX, sulfhydryl oxidases; ALT, alanine aminotransferase; AST, aspartate aminotransferase; TB, total bilirubin; DB, direct bilirubin; ELD, end-stage liver disease; CHB, chronic hepatitis B; CHB-P, CHB with persistently normal ALT levels; ICs, inactive carriers; CI, confidence interval.

First we analyzed serum samples from healthy controls and hepatitis B patients with clinically confirmed ELD, CHB, CHB-P, or ICs, where all of the patients had normal ALT values (< 40 U/L) (Fig. 2A). Using these samples, SOX and DOC activities were determined and compared as biomarkers for disease with AST, DB or TB using ROC curve analyses. It should be noted that the ELD patients had clinically verified irreversible pathological alterations in the liver despite normal ALT levels. The AUC value for DOC in the ELD patients was 0.983, which was significantly higher than that of any of the other examined serum parameters including SOX (0.848), AST (0.865), TB (0.754) and DB (0.808) (P all < 0.0001) (Fig. 2B). The AUC value for AST (0.865) suggests that AST could have some complementary value in this patient cohort, in spite of the lack of abnormal ALT. However, in CHB patients (Fig. 2C) that present less clinical severity, AST had an inadequate AUC value (0.648). So did SOX (0.614), not alone TB (0.528) and DB (0.497). However, DOC was still performing well as a biomarker also in CHB, with an AUC value as high as 0.956 (Fig. 2C), which was significantly superior to all the other potential biomarkers that were examined (P all < 0.0001). DOC also possessed informative AUC values of 0.852 and 0.844 in CHB-P and ICs, respectively, while again the other biomarkers had lost any diagnostic value (Figs. 2D, E). Based on multivariate logistic regression analysis with gender and age as covariates as well as healthy persons as the control, we found that DOC was an strong independent biomarker, with odds ratio values of 12311, 17777, 461 and 493 in subgroups of ELD, CHB, CHB-P and ICs, respectively (P all < 0.0001), while the odds ratio values of the other biomarkers (SOX, AST, TB and DB) were at the range of 0.5-1.7 (Table 1).

**Table 1:** Multivariable logistic regression analysis of biomarkers for chronic hepatitis B patients with normal ALT.

Group	Variable	Odds ratio	95% CI	P value
ELD	DOC	12311	995-152293	< 0.0001
	SOX	1.03	0.99-1.07	0.09
	AST	1.16	1.07-1.25	< 0.001
	TB	0.94	0.86-1.03	0.183
	DB	1.67	1.26-2.20	< 0.001
CHB	DOC	17777	2581-122417	< 0.0001
	SOX	1.01	0.98-1.03	0.678
	AST	1.05	0.99-1.11	0.101
	TB	1.03	0.96-1.10	0.38
	DB	0.90	0.71-1.14	0.364
CHB-P	DOC	461	142-1494	< 0.0001
	SOX	0.97	0.95-1.00	0.037
	AST	1.14	1.08-1.19	< 0.0001
	TB	1.09	1.03-1.16	0.003
	DB	0.7	0.61-0.80	< 0.0001
ICs	DOC	493	151-1608	< 0.0001
	SOX	0.96	0.94-0.98	0.001
	AST	1.11	1.05-1.16	< 0.0001
	TB	1.15	1.08-1.23	< 0.0001
	DB	0.52	0.42-0.64	< 0.0001

Note: Multivariable logistic regression model was adjusted for gender and age.

DOC, dithiothreitol-oxidizing capacity; SOX, sulfhydryl oxidases; ALT, alanine aminotransferase; AST, aspartate aminotransferase; TB, total bilirubin; DB, direct bilirubin; ELD, end-stage liver disease; CHB, chronic hepatitis B; CHB-P, CHB with persistently normal ALT levels; ICs, inactive carriers; CI, confidence interval.

### 3.3 Assessment of sensitivity at 95% specificity in serum from hepatitis B patients with normal ALT levels

**Fig. 3:**
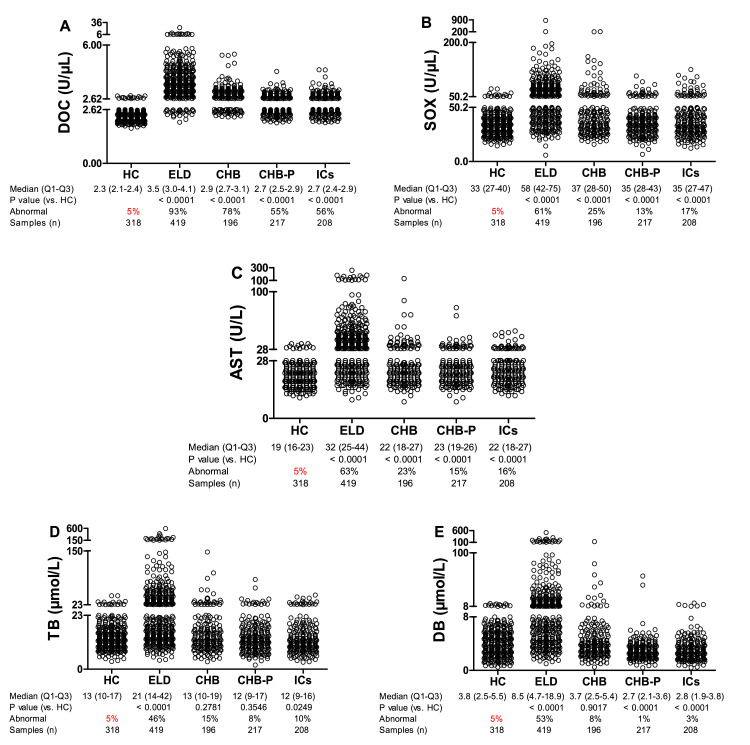
Sensitivity at 95% specificity of serum biomarkers in chronic hepatitis B patients with normal ALT activity. (A-E) DOC, SOX, AST, TB and DB, respectively (Mann Whitney test). Cutoff values of the HC at 95% specificity were 2.62 (U/μL, DOC), 50.2 (U/μL, SOX), 28 (U/L, AST), 23 (μmol/L, TB) and 8 (μmol/L, DB). Abnormal indicates the rate over the cutoff value. Q, quartile; DOC, dithiothreitol-oxidizing capacity; SOX, sulfhydryl oxidases; ALT, alanine aminotransferase; AST, aspartate aminotransferase; TB, total bilirubin; DB, direct bilirubin; HC, healthy controls; ELD, end-stage liver disease; CHB, chronic hepatitis B; CHB-P, CHB with persistently normal ALT levels; ICs, inactive carriers.

Although the hepatitis B patients analyzed above were selected for normal ALT levels, the average ALT levels in the four subgroups were still significantly increased compared to the HC group (P all < 0.0001) (Fig. 2A). We therefore performed repeated analyses for the other biomarkers analyzed here, showing that also DOC, SOX and AST had significantly increased average levels in these patient groups (Figs. 3A-C), but not TB or DB (Figs. 3D, E). Using these data to define limits for 95% specificity, the cutoff values for clinically validated healthy persons (HC) were 2.62 U/μL (DOC), 50.2 U/μL (SOX), 28 U/mL (AST), 23 μmol/L (TB), and 8 μmol/L (DB) (Fig. 3). Using those values as the upper limit for normal, the fraction of the ELD subgroup having higher values than that (referred to herein as sensitivity) was 93% for DOC, whereas none of the other biomarkers had more than 63% (Fig. 3). In the CHB subgroup, the sensitivity for DOC was 78%, whereas the sensitivity for the other biomarkers was no more than 25% (Fig. 3). In the CHB-P and ICs subgroups, presenting with the least severe disease, sensitivity for DOC was 55% and 56%, respectively, while the sensitivity of the other biomarkers was no more than 15% and 17%, respectively (Fig. 3). These results suggest that DOC can be a promising biomarker for assessment of disease in hepatitis B patients presenting with normal ALT values.

### 3.4 DOC exhibits the lowest variation among examined biomarkers

Variation of DOC in healthy people was narrow (CV = 9.4%) (Fig. S1B); whereas variations of the other examined biomarkers including SOX in healthy people were large, among which the narrowest was AST with a CV of 23.1% (Fig. S1D). For reference, the CV of ALT was 37.5% (Fig. S1A). The narrow variation of DOC in healthy people suggests that the homeostasis of DOC is highly regulated. A modest elevation over its cutoff value (2.62 U/μL) may thus potentially indicate hepatic disorder. On the other hand, variation of DOC in each CLD subgroup with normal ALT was also the smallest among tested biomarkers (Fig. S1G). The profile of narrow variation in both healthy persons and each subgroup of the CLD patients with normal ALT contributes to a probability for higher diagnostic accuracy. Since DOC outperformed SOX as a potential biomarker, we next focused on further validating DOC for such use.

### 3.5 DOC in hepatitis B patients does not correlate with ALT

**Fig. 4:**
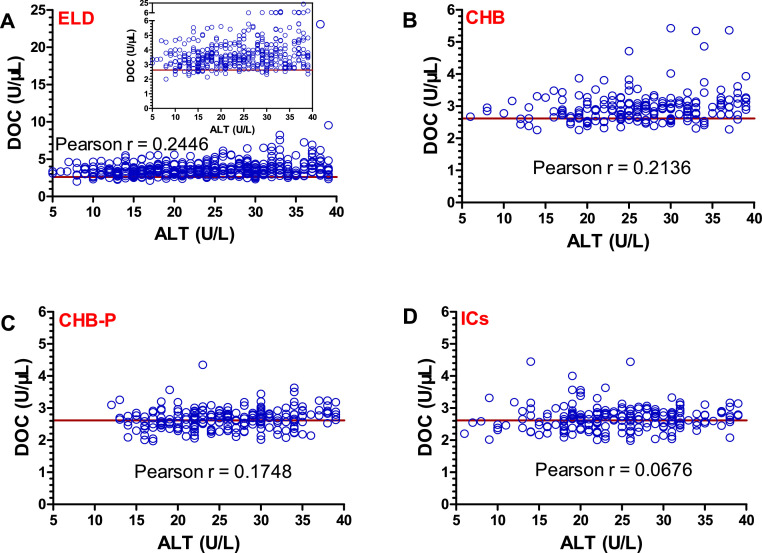
Lack of correlation between DOC and ALT in chronic hepatitis B patients with normal ALT. The graphs show DOC and ALT correlations for patient samples from (A) ELD patients, (B) CHB patients, (C) CHB-P patients, and (D) IC patients. The red line indicates the limit for a normal DOC value with 95% sensitivity, as determined in Fig. 3A. The y-axis is broken in the inserted graph in A to better show DOC distribution over the whole range of ALT levels. DOC, dithiothreitol-oxidizing capacity; ALT, alanine aminotransferase; ELD, end-stage liver disease; CHB, chronic hepatitis B; CHB-P, CHB with persistently normal ALT levels; ICs, inactive carriers.

It has been demonstrated that ALT levels under the revised ULN can not be used to exclude liver disease [5,10]. We thus further analyzed the hepatitis B patients with ALT levels below the revised ULN with regards to their DOC values. For male and female patients, AUC values of DOC were 0.979 and 0.985 (ELD), 0.951 and 0.945 (CHB), 0.820 and 0.894 (CHB-P), and 0.835 and 0.853 (ICs), respectively (Table 2). It should again be noted that ALT levels were low and, by definition, normal in all these patients (<40 U/L), but it was possible that it may still correlate with the DOC values. However, we found that there was an evident lack of correlation between ALT and DOC in all subgroups of these patients (all Pearson r < 0.25), while all presented with a dominance of abnormally high DOC levels irrespective of their ALT levels within this analyzed range (Fig. 4).

**Table 2:** AUC values of DOC for chronic hepatitis B patients with ALT under the revised ULN.

	AUC (95% CI)	
	Male (ALT < 30 U/L)	Female (ALT < 19 U/L)
ELD	0.979 (0.966 - 0.992)	0.985 (0.965 - 1)
CHB	0.951 (0.923 - 0.980)	0.945 (0.889 - 1)
CHB-P	0.820 (0.764 - 0.876)	0.894 (0.808 - 0.980)
ICs	0.835 (0.773 - 0.898)	0.853 (0.772 - 0.934)

AUC, area under the curve; ALT, alanine aminotransferase; ULN, upper limit of normal; ELD, end-stage liver disease; CHB, chronic hepatitis B; CHB-P, CHB with persistently normal ALT levels; ICs, inactive carriers; CI, confidence interval.

We next asked whether DOC correlated with ALT using samples from CHB-associated ELD or CHB patients presenting abnormally high levels of ALT, and assessed the performances of DOC and the other potential biomarkers analyzed here in these patients. Table S4 shows the ROC analyses of the serum markers in CHB-associated ELD or CHB patients having abnormal ALT values. The AUC for DOC in ELD or CHB patients reached 0.996 and 0.987, respectively, which were significantly higher than those of SOX, TB and DB, and non-significantly higher than that of AST, an indispensible biomarker for CLDs. Interestingly, analyzing the possible correlation between DOC and ALT in the samples of ELD or CHB patients presenting abnormally high ALT levels, again we found no evident correlation between the two biomarkers, while the DOC was still elevated above normal for a vast majority of the samples (Fig. S2). This shows that the DOC and ALT should be considered two independent biomarkers for liver disease, as they display no covariance with each other in the patient cohorts studied here.

We further examined the potential association of DOC and liver fibrosis. Fibrosis-associated indicators (APRI and FIB-4) were available from 251 patients (ELD, 96; CHB, 30; CHB-P, 54; and ICs, 71). As shown in Fig. S3, DOC was significantly correlated with APRI (Pearson r = 0.4905, P < 0.0001) or FIB-4 (Pearson r = 0.4421, P < 0.0001).

### 3.6 Assessment of DOC and ALT as biomarkers in AMI and other diseases

**Fig. 5:**
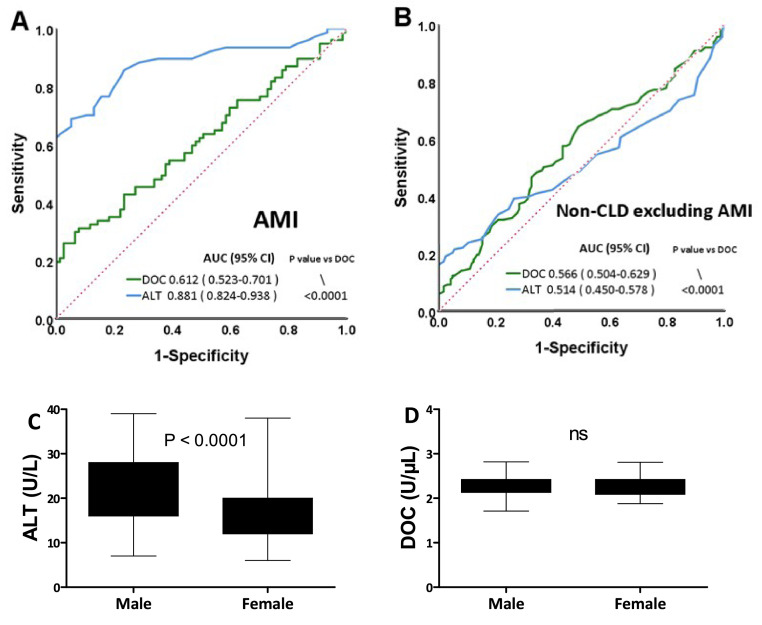
Sensitivity comparisons of ALT and DOC in diseases outside of CLDs as well as gender influence on ALT and DOC. (A) ROC analyses of AMI patients. (B) ROC analyses of patients with non-CLD diseases excluding AMI. (C) Serum ALT of healthy female and male. (D) Serum DOC of healthy female and male. In C and D, Data are plotted as Whiskers: Min to Max (n = 106 and 212 in female and male, respectively. Mann Whitney test). AUC, area under the curve; ROC, receiver operating characteristic; DOC, dithiothreitol-oxidizing capacity; ALT, alanine aminotransferase; AMI, acute myocardial infarction; CLD, chronic liver disease; CI, confidence interval; NS, not significant.

ALT values are often increased in AMI patients [22,23]. We thus next compared the performance of DOC and ALT as biomarkers in AMI patients. The AUC value of a ROC analysis for ALT in AMI was 0.881, which was significantly higher than that for DOC (0.612, P < 0.0001) (Fig. 5A). This shows how ALT increases in serum also correlate well with AMI, as is well known, but that DOC does not seem to be affected to a major extent by AMI. We also examined DOC performance in other patient groups, where ALT is typically not increased, such as stroke, diabetes and pulmonary tuberculosis, revealing similar AUC values for ALT and DOC of 0.514 and 0.566, respectively (P = 0.2253, Fig. 5B). These results suggest that DOC in serum, similar to ALT, is not affected to any major extent in these other diseases. Gender is another confounder affecting ALT sensitivity, with ALT levels generally being higher in healthy males than in healthy females [7], as also found here (Fig. 5C). Unlike ALT, gender seemed not to affect the DOC levels in healthy donors (Fig. 5D).

## 4 Discussion

We have here found that the new assay for DOC measurements yields a serum biomarker for liver disease that shows good promise for use in chronic hepatitis B patients, even irrespective of their ALT values. Most important, with ALT often being normal in several cases of chronic hepatitis, especially in cases with major fibrosis, the DOC biomarker may prove be of significant clinical value as an additional biomarker together with ALT, and should thus be further evaluated for use in monitoring of such patients.

A systematic review with meta-analysis found that approximately one fifth of CHB-P patients has significant hepatic fibrosis, based on liver biopsy findings [10]. ICs account for the largest subgroup among HBV-infected individuals. Up to 30% of all IC patients are likely to undergo spontaneous reactivation of hepatitis B, with increased risk of progressive liver injury or hepatic decompensation [24,25]. Given that we found over 30% sensitivity at 99% specificity for CHB-P or ICs with DOC, and often low ALT levels in these patient groups, DOC could potentially be a powerful biomarker for screening and continued follow-up. If most of these patients with abnormal DOC have been at or finally develop into liver fibrosis stage, progressive liver injury and inflammation, or hepatic decompensation, DOC should be a useful biomarker for predicting long-term outcomes in these two subgroups. Many CHB or HBV-associated ELD patients have normal ALT; however, most of them have abnormal DOC. If a good prognosis is associated with persistent decrease of DOC and visa versa, DOC should be a useful biomarker for predicting long-term outcomes in these two subgroups. Overall, DOC provides a tool for better monitoring of disease, especially in patients exhibiting normal ALT, although DOC cannot discriminate different subgroups of HBV carriers. Future studies focusing on the possible association of DOC with fibroscan and histological score, the gold standard of liver fibrosis, will be warranted. A major concern is whether the high sensitivity of DOC found in the hepatitis B patients also exhibits in other diseases outside of CLDs. We found that DOC was not elevated in the patients with non-liver diseases analyzed here, and it was still insensitive as biomarker in AMI patients where ALT is easily increased, but there may be other diseases or conditions where DOC can be elevated in the absence of liver disease. This should be studied further.

The lack of correlation between DOC and ALT suggests that the two biomarkers increase in serum by different mechanisms in patients having CLDs. It was demonstrated earlier that the enzyme QSOX1 accounts for the major part of human serum thiol oxidation activity, since inhibitory monoclonal antibody specific for human QSOX1 largely inhibits this activity [19]. QSOX1 can be efficiently secreted from mammalian cells and the processing of QSOX1 within the Golgi apparatus affects its secretion [26]. A highly conserved N-linked glycosylation site is required for QSOX1 secretion from mammalian cells [27] and quantitative proteomics have revealed that serum QSOX1 gradually increases with disease advancement caused by HBV [28]. The physiological function of extracellular QSOX1 has also been characterized and suggested to relate to remodelling of collagens in the extracellular matrix. [29], [30], [31] Moreover, the production of hydrogen peroxide by QSOX1 in the extracellular space may trigger inflammation [32]. With modulation of extracellular matrix being a hallmark of liver fibrosis and with hepatic stellate cells and portal fibroblasts being important sources of matrix proteins in hepatic fibrosis [33,34], it is possible that secreted QSOX1 is related to physiological responses to CLD.

An important question is why the DOC assay shows better performance than the SOX assay, as both assays measure QSOX1-like activity. One reason may be H_2_O_2_ metabolizing enzymes in serum such as catalase or glutathione peroxidase (GPx3) that could potentially show activity in the SOX assay. Such H_2_O_2_ metabolizing enzymes would, however, not interfere with the DOC assay, which could thereby explain its better performance compared to the SOX assay.

In the present study, the median age in ELD subgroup was significantly higher than in the HC group, which may potentially be a limitation. However, we found no evident correlation between DOC and age in any examined groups (Pearson r: -0.13-0.17), and neither were the DOC levels in healthy persons affected by gender. Still, the DOC assay needs to be further evaluated in other diseases than those studied herein, including liver diseases and non-liver diseases. A limitation of DOC is that it can not discriminate different subgroups of HBV carriers. Other limitations to our present study include the lack of information on fibrotic scores or histopathological findings in correlation to DOC values in individual patients. Such potentially important determinants underlying increased DOC levels should be addressed in forthcoming studies, as should the molecular mechanisms of DOC secretion and, finally, the possible biological functions of this enzymatic activity in serum.

Based upon the results of this exploratory pilot study, we suggest that DOC should be considered as a new seemingly reliable complementary biomarker for monitoring of disease in chronic hepatitis B patients that can be used together with ALT for improved diagnostic power. Additional studies to corroborate the finding are needed prior to its application.

## Data sharing statement

All data generated or analyzed during this study are included in this article and its supplementary material files. Further enquiries can be directed to the corresponding authors.

## Contributors

JZ conceived and supervised the study, wrote the initial draft, and coordinated with all other co-authors in writing of the final version of the manuscript. LY and KZ contributed experimentally by measuring the DOC and SOX activities in all serum samples, and assisted in the writing process. YZ, ZL, TH, XZ, LL, and ZZ: contributing clinicians with patient contacts, responsible for clinical data, and overseeing collections of serum samples. In addition, ZZ participated in data analysis and interpretations. EA contributed by discussing experimental layout and interpretations, and helped writing the manuscript. JZ and ZZ accessed and were responsible for the raw data associated with the study.

## Declaration of Competing Interest

JZ has a Chinese Patent associated with measurement of total thiol-oxidizing capacity in serum. The other authors declare no competing interests.

## Appendix Supplementary materials

Image, application 1
