# Supplementary material for: Evaluation of dithiothreitol-oxidizing capacity (DOC) as a serum biomarker for chronic hepatitis B in patients exhibiting normal alanine aminotransferase levels: a pilot study towards better monitoring of disease
Source: eClinicalMedicine. 2021 Oct 30;42:101180. doi: 10.1016/j.eclinm.2021.101180 (PMC8569636; doi:10.1016/j.eclinm.2021.101180)
Supplement: Supplementary file 1 [file mmc1.doc]

**Evaluation of dithiothreitol-oxidizing capacity (DOC) as a serum biomarker for chronic hepatitis B in patients exhibiting normal alanine aminotransferase levels: a pilot study towards better monitoring of disease**

Lumin Yang, Yafei Zhang, Ke Zhang, Zhongping Liu, Tengfei He, Xiaowei Zheng, Lei Li, Elias S J Arnér, Zhenhua Zhang, Jinsong Zhang

**Captions for Supplementary Material**

Table S1 Demographic features of HC and chronic hepatitis B patients

Table S2 Demographic features of HC and AMI patients or non-CLD patients excluding AMI

Table S3 Demographic features of HC and chronic hepatitis B patients with ALT under the revised ULN

Table S4 ROC analyses of serum biomarkers in CHB-associated ELD or CHB patients with abnormal ALT activity

Figure S1 Coefficient of variation of various serum parameters

Figure S2 Lack of correlation between DOC and ALT in chronic hepatitis B patients having abnormally high ALT values

Figure S3 Correlation between DOC and fibrosis score in chronic hepatitis B patients with normal ALT

**Table S1 Demographic features of HC and chronic hepatitis B patients**

| Variable | Age (years)  Median  (interquartile range) | No. (%) | |
| --- | --- | --- | --- |
|  |  | Male | Female |
| ALT < 40 U/L | | | |
| HC | 42 (30-53) | 212 (67) | 106 (33) |
| ELD | 53 (47-63)*** | 335 (80) | 84 (20) |
| CHB | 40 (31-49) | 113 (58) | 83 (42) |
| CHB-P | 41 (32-50) | 151 (70) | 66 (30) |
| ICs | 41 (31-54) | 104 (50) | 104 (50) |
| ALT ≥ 40 U/L | | | |
| ELD | 51 (44-62)*** | 283 (84) | 55 (16) |
| CHB | 38 (29-47) | 239 (76) | 76 (24) |

***, P < 0.0001, compared to HC (Mann Whitney test)

ALT, alanine aminotransferase; HC, healthy controls; ELD, end-stage liver disease; CHB, chronic hepatitis B; CHB-P, CHB with persistently normal ALT levels; ICs, inactive carriers.

**Table S2 Demographic features of HC and AMI patients or non-CLD patients excluding AMI**

|  | Age (years)  Median  (interquartile range) | No. (%) | |
| --- | --- | --- | --- |
|  |  | Male | Female |
| HC | 60 (55-65) | 48 (62) | 29 (38) |
| AMI | 68 (62-76)*** | 48 (62) | 29 (38) |
|  | | | |
| HC | 47 (36-54) | 105 (64) | 58 (36) |
| Non-CLD excluding AMI | 57 (47-69)*** | 105 (64) | 58 (36) |

***, P < 0.0001, compared to the HC (Mann Whitney test)

HC, healthy controls; AMI, acute myocardial infarction; CLD, chronic liver disease.

**Table S3 Demographic features of HC and chronic hepatitis B patients with ALT under the revised ULN**

|  | Age (years)  Median (interquartile range) (n) | |
| --- | --- | --- |
|  | Male (ALT < 30 U/L) | Female (ALT < 19 U/L) |
| HC | 44 (31-53) (165) | 37 (30-53.5) (73) |
| ELD | 53 (47-62)*** (222) | 61 (44-67)*** (41) |
| CHB | 44 (34-50) (61) | 33 (25.5-47) (17) |
| CHB-P | 42 (32-51) (89) | 39 (32-49.25) (18) |
| ICs | 50.5 (35-57.5) (66) | 44.5 (30-55.25) (28) |

***, P < 0.0001, compared to HC (Mann Whitney test)

ALT, alanine aminotransferase; ULN, upper limit of normal; HC, healthy controls; ELD, end-stage liver disease; CHB, chronic hepatitis B; CHB-P, CHB with persistently normal ALT levels; ICs, inactive carriers.

Table S4 ROC analyses of serum biomarkers in CHB-associated ELD or CHB patients with abnormal ALT activity

| Biomarker | AUC (95% CI) | compared to DOC |
| --- | --- | --- |
| **ELD (338) vs. HC (318)** | | |
| DOC | 0.996 (0.992 to 1) |  |
| SOX | 0.946 (0.928 to 0.963) | <0.0001 |
| AST | 0.991 (0.982 to 0.999) | 0.2462 |
| TB | 0.892 (0.866 to 0.917) | <0.0001 |
| DB | 0.914 (0.893 to 0.935) | <0.0001 |
| **CHB (315) vs. HC (318)** | | |
| DOC | 0.987 (0.981 to 0.994) |  |
| SOX | 0.733 (0.695 to 0.772) | <0.0001 |
| AST | 0.981 (0.973 to 0.989) | 0.2256 |
| TB | 0.624 (0.581 to 0.668) | <0.0001 |
| DB | 0.635 (0.592 to 0.678) | <0.0001 |

Note: Ages were 51 (44-62), 38 (29-47) and 42 (30-53) in the ELD, CHB and HC groups, respectively. Significant difference occurred between the HC and ELD (P < 0.0001). Male accounted for 82%, 69% and 67% in the ELD, CHB and HC groups, respectively.

AUC, area under the curve; ROC, receiver operating characteristic; DOC, dithiothreitol-oxidizing capacity; SOX, sulfhydryl oxidases; ALT, alanine aminotransferase; AST, aspartate aminotransferase; TB, total bilirubin; DB, direct bilirubin; HC, healthy controls; ELD, end-stage liver disease; CHB, chronic hepatitis B; CI, confidence interval.

**Fig. S1. Coefficient of variation of various serum parameters.** (A-F) CV of serum biomarkers in healthy persons (n = 318). Data are plotted as Whiskers: Min to Max. (G) CV of serum biomarkers in chronic hepatitis B patients with normal ALT. CV, coefficient of variation; DOC, dithiothreitol-oxidizing capacity; SOX, sulfhydryl oxidases; ALT, alanine aminotransferase; AST, aspartate aminotransferase; TB, total bilirubin; DB, direct bilirubin; ELD, end-stage liver disease; CHB, chronic hepatitis B; CHB-P, CHB with persistently normal ALT levels; ICs, inactive carriers.

**Fig. S2. Lack of correlation between DOC and ALT in chronic hepatitis B patients having abnormally high ALT values.** The correlation plots illustrate the DOC and ALT values in samples from (A) ELD patients and (B) CHB patients, having ALT values above 40 U/L. The red line indicates the limit for a normal DOC value with 95% sensitivity, as determined in Fig. 3A. The x-axes and y-axes are broken in inserted graphs in order to better show DOC distribution over the whole range of ALT levels. DOC, dithiothreitol-oxidizing capacity; ALT, alanine aminotransferase; ELD, end-stage liver disease; CHB, chronic hepatitis B.

**Fig. S3. Correlation between DOC and fibrosis score in chronic hepatitis B patients with normal ALT.** 251 chronic hepatitis B patients (ELD, 96; CHB, 30; CHB-P, 54; and ICs, 71) with APRI and FIB-4 data were used for the analyses. (A) APRI. (B) FIB-4. DOC, dithiothreitol-oxidizing capacity; ALT, alanine aminotransferase; APRI, aspartate aminotransferase-to-platelet ratio index; FIB-4, fibrosis-4; ELD, end-stage liver disease; CHB, chronic hepatitis B; CHB-P, CHB with persistently normal ALT levels; ICs, inactive carriers.
